# Technostress, Coping, and Anxious and Depressive Symptomatology in University Students During the COVID-19 Pandemic

**DOI:** 10.5964/ejop.4725

**Published:** 2022-08-31

**Authors:** John Galvin, Michael Scott Evans, Kenisha Nelson, Gareth Richards, Eirini Mavritsaki, Theodoros Giovazolias, Katerina Koutra, Ben Mellor, Maria Clelia Zurlo, Andrew Paul Smith, Federica Vallone

**Affiliations:** 1 Birmingham City University, Birmingham, United Kingdom; 2 Cardiff University, Cardiff, United Kingdom; 3 University of Technology, Kingston, Jamaica; 4 Newcastle University, Newcastle upon Tyne, United Kingdom; 5 University of Crete, Rethymno, 74100, Greece; 6 University of Naples Federico II, Naples, Italy; Creighton University, Omaha, NE, USA

**Keywords:** university students, technostress, coping, anxiety, depression, COVID-19

## Abstract

The COVID-19 pandemic raised many challenges for university staff and students, including the need to work from home, which resulted in a greater reliance on technology. We collected questionnaire data from university students (N = 894) in three European countries: Greece, Italy, and the United Kingdom. Data were collected between 7th April 2020 and 19th June 2020, representing a period covering the first lockdown and university closures in these countries and across Europe generally. We tested the hypotheses that technology-related stressors (techno-overload, work-home conflict, techno-ease, techno-reliability, techno-sociality, and pace of change) would be associated with anxiety and depressive symptoms, and that coping styles (problem-focused, emotion-focused, and avoidance) would mediate these relationships. Results showed significant positive associations between techno-overload, work-home conflict and anxiety and depressive symptoms, and significant negative associations between techno-reliability, techno-ease and anxiety and depressive symptoms. A significant negative association was found between techno-sociality and depressive symptoms but not anxiety symptoms. No evidence was found for an association between pace of change and anxiety or depressive symptoms. Multiple mediation analyses revealed significant direct effects of techno-overload, work-home conflict and techno-ease on anxiety symptoms, and of work-home conflict and techno-ease on depressive symptoms. Work-home conflict had significant indirect effects on anxiety and depressive symptoms through avoidance coping. Techno-overload and techno-ease both had significant indirect effects on anxiety symptoms through problem- and emotion-focused coping. Techno-ease also had a significant indirect effect on depressive symptoms through problem-focused coping. The findings add to the body of evidence on technostress amongst university students and provide knowledge on how technostress translates through coping strategies into anxious and depressive symptoms during the disruption caused by the outbreak of a pandemic disease.

In response to the novel coronavirus 2019 (COVID-19), the World Health Organisation declared a global pandemic on 11th March 2020. As of 15th May 2021, the Coronavirus Resource Centre at Johns Hopkins University and Medicine (2021) reported 161,566,026 confirmed cases and 3,353,630 deaths worldwide. Government officials and public health experts have taken several steps to control the spread of the virus, including imposing special measures on their populations, such as self-isolation and restriction of movement and assembly, which have led to a high number of individuals having to work from home.

The higher education (HE) sector has been severely impacted by the COVID-19 crisis. The pandemic necessitated a rapid transition from a predominantly face-to-face teaching model to an online only or heavily blended learning model for many academic courses (Watermeyer et al., 2021). Although online teaching and learning is not new for many universities, a predominantly online model is new to many staff and students. This transition to a purely digital teaching and learning experience has, by its very nature, an intrinsic expectation that staff and students are able to use technology for all intents and purposes, that the technology is reliable, and that they have workspaces at home which would mirror the workplace environment, i.e., without distractions or conflicting home demands (Sahu, 2020).

Technostress was first defined by Brod (1984) as the inability to adapt or cope with information and communication technologies (ICTs) in a healthy manner. This definition is in line with Lazarus and Folkman’s (1984) suggestion that stress refers to any demand, event or situation that disturbs the adaptive state and threatens to exceed the individual’s resources and skills. If the individual’s adaptive state is altered by an event, this may provoke a coping response (Lazarus & Folkman, 1991). If people maintain adaptive coping responses, they are less likely to appraise a situation as threatening and have improved mental health outcomes (Freire et al., 2016; Taylor & Stanton, 2007).

Previous research has shown that technostress in university students is associated with a range of psychopathological outcomes including higher anxiety, depression, burnout, and suicidality (Kim et al., 2006; Wang et al., 2020). Several factors have been identified as determinants of technostress (hereinafter referred to as *techno-stressors*) (Ayyagari et al., 2011; DeLone & McLean, 2003; Jiang et al., 2002; Kreiner, 2006; Moore, 2000; Moore & Benbasat, 1991; Netemeyer et al., 1996; Weiss & Heide, 1993). *Techno-overload* refers to the situation in which individuals feel forced by ICTs to work faster and longer. *Work-home conflict* is when work and private life merge due to ICT usage. *Pace of change* refers to an individual’s perception of frequent ICT-related changes and upgrades. *Techno-ease* refers to whether or not the user feels competent enough to use ICTs and to achieve the desired results. *Techno-sociality* refers to ICT as a social communication tool by which individuals can contact, or be contacted by other people. Finally, *techno-reliability* is the perception of the consistency or dependability of ICTs.

An ability to cope with techno-stressors will depend on individual resources (e.g., coping competencies) as well as environmental factors (e.g., circumstances). Coping can be defined as acts of adaptation that an individual performs in response to events that occur in his/her environment (Folkman & Lazarus, 1988; Lazarus & Folkman, 1984). Coping responses are commonly categorised into three broader themes: problem-focused, emotion-focused, and avoidance coping (Lazarus & Folkman, 1984; Roth & Cohen, 1986). Problem-focused coping involves handling the stressor by taking action to solve the problem, facing it head-on, and making attempts to resolve the underlying cause. Examples of problem-focused coping include planning and taking active steps to address the problem. Emotion-focused coping involves the regulation of feelings and emotional responses that arise, as opposed to directly addressing the problem. Examples of emotion-focused coping include accessing social support networks and venting about the problem. Finally, avoidance coping is characterised by coping efforts aimed at avoiding the stressor, and examples include disengagement, denial, and substance use. While problem-focused coping is often considered the most effective coping strategy and avoidance coping the least effective, research shows that the most effective strategy can depend on the type of stressor encountered and/or other environmental circumstances (Bonanno & Burton, 2013; Lee-Baggley et al., 2005). Therefore, individuals might not differ only in their choice of coping strategies, but also in the extent and context in which they engage in any single strategy.

The digitalisation of society and the labour-market has been on a constant rise over the last decades (Vasilescu et al., 2020), and this shift has been exacerbated by the COVID-19 crisis. Although digitalisation has some advantages, it also results in important challenges, including a rise in the phenomenon of technostress in distance education. It has thus never been more pertinent to investigate techno-stressors and their relationships with mental health outcomes in the student population. In this study, we explored the relationships between techno-stressors, coping strategies and anxious/depressive symptoms in a sample of students from three European countries: Greece, Italy and UK. It was hypothesised that:

*H1*: Techno-overload, work-home conflict and pace of change would be positively associated with anxiety and depressive symptoms.

*H2*: Techno-ease, techno-reliability and techno-sociality would be negatively associated with anxious and depressive symptoms.

*H3*: Coping style would mediate the association between techno-stressors and anxious/depressive symptoms.

## Method

### Procedure and Participants

Participants were given an information sheet that provided basic details of the study, and were required to complete a consent form before taking part. An online cross-sectional survey (hosted by Qualtrics) was distributed to university students in Greece, Italy and the UK between 7th April 2020 and 19th June 2020. This period covered a timeframe in which the first lockdown was implemented and included full closures of universities in all three countries. Participants were studying at undergraduate or masters level, and were recruited from the affiliative institutions of the authors through research participation databases and student learning forums. In Greece, a link to the survey was sent by e-mail to faculty members in universities across different cities and regions of the country (Crete, Athens, Thessaloniki, Thessaly, Epirus, Thrace) who then forwarded it to their students using either academic mailing lists or student social media groups. In Italy, a link to the survey was sent via academic mailing lists and social media groups for three universities in the southwestern region of Campania (Naples and Benevento). In the UK, the Psychology Department Research Participation Schemes (RPS) at Birmingham City University and Newcastle University were used. The questionnaire link was also sent to student social media groups at Birmingham City University, Newcastle University, Northumbria University, and the University of Liverpool. Students recruited in the UK through RPS were awarded participation credits. All other participants did not receive any reward for completing the study.

### Materials

After demographic questions (sex, age, relationship status, course status, level of study and employment status), a series of questions on technology usage was presented. This included a question asking the participants to provide detail on the technological device(s) they have in their home, as well as a question on the device(s) that they personally own. Participants were also asked how many people (including themselves) live in their household, and whether they have their own personal space to use technological device(s).

#### Technostress Scale

Techno-stressors were measured with validated survey items from prior studies. The constructs, items, and internal reliability coefficients for the present study are detailed in Table 1. Participants responded to 17 items on a seven-point Likert scale (1 = Strongly Disagree to 7 = Strongly Agree).

**Table 1:** Technostress Constructs, Items, and Cronbach’s Alpha Scores for the Present Study

Technostress Factors and *Items*	Reference(s)	Overall Sample	Greece	Italy	UK
Techno-Overload					
ICTs create many more requests and problems than I would otherwise experience I feel busy or rushed due to ICTs I feel pressured due to ICTs	Moore (2000)	α = .82	α = .83	α = .78	α = .84
Work-Home Conflict					
Using ICTs blurs boundaries between my work and my home life Using ICTs for work related responsibilities creates conflicts with my home responsibilities I do not get everything done at home because I find myself completing work due to ICTs	Kreiner (2006)	α = .77	α = .74	α = .78	α = .80
Techno-Ease					
Learning to use ICTs is easy for me ICTs are easy to use It is easy to get results that I desire from ICTs	Moore and Benbasat (1991)	α = .85	α = .86	α = .82	α = .85
Techno-Reliability					
The features provided by ICTs are dependable The capabilities provided by ICTs are reliable ICTs behave in a highly consistent way	DeLone and McLean (2003) Jiang et al. (2002)	α = .85	α = .86	α = .82	α = .85
Techno-Sociality					
The use of ICTs enables others to have access to me The use of ICTs enables me to be in touch with others	Ayyagari et al. (2011)	α = .74	α = .59	α = .90	α = .86
Pace of Change					
I feel that there are frequent changes in the features of ICTs I feel that the capabilities of ICTs change often I feel that the way ICTs work changes often	Weiss and Heide (1993)	α = .84	α = .85	α = .88	α = .83

#### Coping Style

The 60-item version of the COPE inventory (Carver et al., 1989) was used to measure coping style. It comprises 15 subscales: positive reinterpretation and growth, mental disengagement, focus on and venting of emotions, use of instrumental social support, active coping, denial, religious coping, suppression of competing activities, humour, behavioural disengagement, restraint, use of emotional social support, substance abuse, acceptance, and planning. Although the original scale has 15 subscales, Carver et al. (1989) suggest three higher order factors (problem-focused, emotion-focused, and avoidance coping) based on factor analysis. Overall internal consistency for the COPE factors in the present study were as follows: problem-focused coping α *=* .908; emotion-focused coping α *=* .850; avoidance coping α *=* .702. In Greece, problem-focused coping α *=* .878, emotion-focused coping α *=* .841, and avoidance coping α *=* .716. In Italy, problem-focused coping α *=* .844, emotion-focused coping α *=* .821, and avoidance coping α *=* .669. In the UK, problem-focused coping α *=* .941, emotion-focused coping α *=* .861, and avoidance coping α *=* .711.

#### Anxious and Depressive Symptoms

The Hospital Anxiety and Depression Scale (Zigmond & Snaith, 1983) consists of 14 items, with seven measuring anxiety symptoms, and seven measuring depressive symptoms. Participants’ responses are coded on a scale of 0–3 for each item. The questionnaire is designed to assess an individual’s mental state over the previous two weeks. Overall internal consistency was α *=* .822 for anxiety symptoms and α *=* .688 for depressive symptoms. In Greece, α *=* .797 for anxiety symptoms and α *=* .673 for depressive symptoms. In Italy, α *=* .818 for anxiety symptoms and α *=* .653 for depressive symptoms. In the UK, α *=* .843 for anxiety symptoms and α *=* .697 for depressive symptoms.

#### Translation of Scales Into Greek and Italian

The UK sample completed the questionnaire in English, including the original English versions of the COPE (Carver et al., 1989) and HADS (Zigmond & Snaith, 1983). For distribution in Italy and Greece, the information sheet, consent form, debrief form, demographic and technostress items were translated into Greek by authors TG, KK, and EM, and into Italian by author FV. The scales were then back-translated into English by the same authors. We used the Italian versions of the COPE (Sica et al., 2008) and HADS (Costantini et al., 1999) in Italy, and the Greek versions of the COPE (Roussi, 2001) and HADS (Michopoulos et al., 2008) in Greece.

### Data Analysis

Data were analysed using JASP software version 0.14.1 (JASP Team, 2020) and statistical significance was set at 5% (two-tailed). Differences between countries on demographic and study variables were examined with ANOVA (Bonferroni corrected) and with chi-square test for categorical variables. Because the utilisation and effectiveness of coping strategies can rely on specific environmental contexts (Bonanno & Burton, 2013; Lee-Baggley et al., 2005), and given the uniqueness of the pandemic situation, we identified coping factors with an exploratory factor analysis (EFA) using principal components extraction and promax oblique rotation. As the technostress scale has not previously been validated in Greek or Italian, we conducted confirmatory factor analysis (CFA) on the scale followed by multi-group confirmatory factor analysis (MGCFA) to examine measurement invariance.

Measurement invariance comprises configural, metric and scalar invariance. Configural invariance examines whether the measurement scale has a similar factor structure across the different countries. It is tested by imposing the same structure across groups and allowing all model estimated parameters to differ. Metric invariance examines whether the rating scales are used similarly in the different countries. It is tested by examining whether the different countries have the same factor loadings for the same item. Finally, scalar invariance examines whether the different countries have the same item intercepts. It is achieved by constraining intercepts to be equal across groups. Establishing scalar invariance would enable meaningful comparison of the means across the countries (Little, 1997). The goodness of fit indices for CFA and MGCFA models include the chi-square (χ^2^) statistic, root mean square error of approximation (RMSEA), comparative fit index (CFI), Tucker-Lews index (TLI), and Standardised Root Mean Squared Residual (SRMR). The common guidelines for an acceptable model fit are: χ^2^, *p* > .05, RMSEA < .08; CFI > .90; TLI > .90; SRMR < .09. As the chi-square test is strongly influenced by sample size (Cheung & Rensvold, 2002), we relied on the RMSEA, CFI, TLI and SRMR to assess model fit. The assessment of measurement invariance involved testing the deterioration of the model fit between the configural, metric and scalar model. Changes in CFI, TLI, and RMSEA of < .01 are considered acceptable (Rutkowski & Svetina, 2014).

We examined the associations between all variables using Pearson’s correlation. This was followed by four multiple linear regression analyses (enter method). The first two regressions included the technostress factors as predictors and HADS anxiety and depression subscale scores as outcomes. The remaining two regressions included COPE factors as predictors and anxiety/depression as outcomes. The independent errors assumption was checked with the Durbin-Watson statistic, and the multicollinearity assumption was tested with Variance Inflation Factor (VIF). Mediation analysis was then performed (bootstrap 5000 iterations and bias-corrected). The predictor variables included in the analysis were each of the significant techno-stressors from the multiple regression step. Mediators were each of the significant COPE factors from the multiple regression step. Outcome variables were the HADS anxiety and depression subscale scores. The maximum likelihood estimation was used to estimate the direct and indirect effects. Background confounders included age, sex (female), relationship status (single), level of study (masters), international student (yes) and employment status (employed). The full information maximum likelihood (FIML) estimation was used to deal with the missing values (< 10%) in the final sample.

## Results

### Demographics

The questionnaire was accessed by *N* = 963 participants. Forty were removed from the analysis as they did not respond to any of the study variables. A further 17 were removed because they reported that they were not students and 12 were removed as they were doctoral level students. This resulted in a total sample size of *N* = 894 (Greece, *n* = 343; Italy, *n* = 120; UK, *n* = 431). Participants were studying a range of subjects, including psychology (*n* = 262), core sciences (biology, chemistry or physics) (*n* = 142), engineering (*n* = 88), medicine (*n* = 83), social studies (*n* = 45), business (*n* = 66), languages (*n* = 42), education (*n* = 36), history (*n* = 36), art or media studies (*n* = 15), geography (*n* = 15), maths (*n* = 15), nursing (*n* = 13), law (*n* = 11), philosophy (*n* = 10), architecture (*n* = 6), and archaeology (*n* = 6). Participants differed significantly across countries on all demographic variables except for sex, ownership of a mobile phone, and having a desktop or mobile phone at home (Table 2).

**Table 2:** Demographic Information For the Overall Sample and Stratified By Nation

Sample Characteristic	Total Sample	Greece	Italy	United Kingdom	Statistic
Sex *n* (%)					
Females	686(77)	267(78)	96(80)	323(75)	
Males	206(23)	74(21)	24(20)	108(25)	χ^2^ = 5.178
Other	0(0)	0(0)	0(0)	0(0)	*p* = .270
Prefer not to say	2(0)	2(1)	0(0)	0(0)	
Age in years					
*M, SD*	21.58, 4.29	22.99, 5. 58	22.47, 4.10	20.20, 2.21	*F* = 46.371
*(Range)*	(18–56)	(18–56)	(19–38)	(18–44)	*p < .*001
Relationship *n* (%)					
Single	747(84)	58(17)	47(39)	42(10)	χ^2^ = 59.229
Relationship	147(16)	285(83)	73(61)	389(90)	*p* < .001
Course *n* (%)					
Full-time	824(96)	318(98)	92(80)	414(99)	χ^2^ = 100.917
Part-time	33(4)	6(2)	23(20)	4(1)	*p* < .001
Study level *n* (%)					
Bachelors	727(89)	264(93)	101(87)	362(87)	χ^2^ = 7.353
Masters	91(11)	20(7)	15(13)	56(13)	*p* = .025
Employment *n* (%)					
Full-time	68(7)	34(11)	8(7)	21(5)	
Part-time	228(27)	27(9)	27(23)	174(40)	χ^2^ = 99.036
Not employed	538(63)	229(77)	83(68)	226(53)	*p* < .001
Prefer not to say	22(3)	10(3)	2(2)	10(2)	
Technology devices at home *n* (%)					
Laptop					
Yes	840(94)	302(88)	110(92)	428(99)	χ^2^ = 43.932
No	54(6)	41(12)	10(8)	3(1)	*p* < .001
Desktop					
Yes	340(38)	118(34)	53(44)	169(39)	χ^2^ = 4.088
No	554(62)	225(66)	>67(56)	262(61)	*p =* .130
Tablet					
Yes	472(53)	142(41)	56(47)	274(64)	χ^2^ = 39.771
No	422(47)	201(59)	64(53)	157(36)	*p* < .001
Mobile					
Yes	841(99)	295(99)	117(98)	429(99)	χ^2^ = 43.932
No	8(1)	3(1)	3(2)	2(1)	*p* = .123
Other^a^	37(4)	8(2)	7(5)	22(5)	—
Technology devices personally owned *n* (%)					
Laptop					
Yes	722(86)	268(78)	95(80)	409(95)	χ^2^ = 51.611
No	122(14)	75(22)	25(20)	22(5)	*p* < .001
Desktop					
Yes	106(12)	50(15)	18(15)	38(9)	χ^2^ = 7.375/
No	788(88)	293(85)	102(85)	393(91)	*p* = .003
Tablet					
Yes	278(31)	97(28)	25(20)	156(36)	χ^2^ = 12.398
No	616(69)	246(72)	95(80)	275(64)	*p* = .002
Mobile					
Yes	842(99)	295(99)	119(99)	428(99)	χ^2^ = 0.208
No	7(1)	3(1)	1(1)	3(1)	*p =* .901
Other^a^	20(2)	4(1)	3(2)	13(3)	—
Personal space? *n* (%)					
Yes	698(79)	254(75)	100(85)	344(80)	χ^2^ = 6.929
No	187(21)	87(25)	19(15)	81(20)	*p =* .031
Number of people living in household *M*	3.58(1.51)	2.87(1.58)	3.69(1.11)	3.85(1.58)	*F* = 40.364
(*SD*)					*p* < .001

*^a^* Responses to “other” included: Game Consoles (*n* = 18), Smart TVs (*n* = 10), Home Hubs (*n* =7), and Smartwatch (*n* = 2).

### EFA on the COPE Scale

Principal components analysis (PCA) was conducted on the COPE scale. The PCA confirmed three factors with eigenvalues greater than 1, which together explained 62% of the variance (Table 3). The factors were aligned closely with the findings of Carver et al. (1989) and represented problem-focused, emotion-focused, and avoidance coping. The first factor represented problem-focused coping, with high loadings from the following COPE subscales: *positive reinterpretation and growth, active coping, restraint, acceptance, humour, suppression of competing activities,* and *planning*. The second factor represented emotion-focused coping, with high loadings from the subscales: *focusing on and venting of emotions, instrumental social support,* and *use of social support.* The third factor represented avoidance coping, with high loadings from the subscales: *denial, substance use, behavioural disengagement, and mental disengagement. Religious coping* did not load highly on any of the three factors. As religion is not a specific focus of our study, the decision was made to exclude this subscale from further analysis.

**Table 3:** Exploratory Factor Analysis of the COPE Subscales

EFA COPE subscales	Factor 1	Factor 2	Factor 3
Planning	.924		
Positive reinterpretation and growth	.859		
Active coping	.849		
Acceptance	.733		
Suppression of competing activities	.720		
Restraint	.626		
Humour	.430		
Use of emotional support		.943	
Instrumental social support		.780	
Focus on and venting of emotions		.749	
Behavioural disengagement			.856
Denial			.677
Substance use			.550
Mental disengagement			.352
Religious coping			

*Note*. Factor loadings below 0.3 are excluded.

### CFA and MGCFA on Technostress Scale

Next, we conducted a series of confirmatory factor analyses on the technostress scale (Table 4). RMSEA, CFI and SRMR values indicate acceptable model fit for the Greek and the UK samples. TLI indicated acceptable fit for the UK sample and was very close to the acceptable threshold for the Greek sample (.893). RMSEA, CFI, TLI and SRMR indicated an insufficient fit for the Italian sample.

**Table 4:** Fit Indices For Technostress

Sample	RMSEA	CFI	TLI	SRMR
Overall	.071 [.065-.077]	.935	.915	.058
Greece	.081 [.070-.091]	.918	.893	.062
Italy	.124 [.107-.142]	.835	.785	.097
UK	.071 [.061-.081]	.940	.921	.062

*Note*. Sub-scales from the confirmatory factor analyses.

To see if the model-data fit could be improved we inspected the modification indices for each country separately. We based a selected model on the UK data, since English is the source language of the scales. Further estimations indicated that deleting the third item on the techno-ease scale *‘it is easy to get results that I desire from ICTs’* increased the fit in all countries. The item was not distinctive enough and cross-loaded with items on the techno-reliability scale. RMSEA, CFI, TLI and SRMR values indicated acceptable fit for the overall sample as well as for each country in the revised model (Table 5). These results provided a good starting point for the subsequent multi-group confirmatory factor analyses.

**Table 5:** Revised CFA

Sample	RMSEA	CFI	TLI	SRMR
Overall	.047 [.040-.055]	.972	.963	.038
Greece	.067 [.055-.079]	.944	.925	.053
Italy	.074 [.051-.096]	.941	.921	.064
UK	.045 [.032-.058]	.977	.968	.041

*Note*. With the removal of Item 3 from the Techno-Ease Scale.

The MGCFA consisted of three steps. The configural equivalent model was estimated first, in which we imposed the same factor structure on the scores in each country. A sufficiently good fit was found (Table 6), suggesting the measurement scale has a similar factor structure across the three countries. Next, we imposed the factor loadings to be the same across countries (Table 6). We expected a slight decrease in fit, which was confirmed, with a RMSEA of .062 and SRMR of .057. However, these are both still above the acceptable thresholds. Finally, we tested the full scalar invariant model and found this was acceptable with ΔRMSEA, ΔCFI, and ΔTLI < .01 (Rutkowski & Svetina, 2014). The comparison of latent means for the techno-stress factors can therefore be justified (Table 6).

**Table 6:** Multi-Group Confirmatory Factor Analysis For the Techno-Stress Scale

MGCFA	RMSEA	CFI	TLI	SRMR	Model comparison	ΔRMSEA	ΔCFI	ΔTLI	ΔSRMR
M1: Configural invariance	.061 [.051-.070]	.961	.947	.049					
M2: Metric invariance	.062 [.053-.070]	.956	.945	.057	M1	.001	.005	.002	.008
M3: Scalar invariance	.069 [.060-.077]	.947	.937	.057	M2	.007	.009	.008	.000

### Differences Between Countries on the Study Variables

Table 7 details the means, standard deviations, and results of the ANOVA and Bonferroni post hoc tests. Significantly higher levels of anxiety and depression were found in the UK sample compared to the other countries. Work-home conflict was significantly higher in the UK compared to Italy. Techno-ease was lower in the Italian sample compared to the other countries, and pace of change was higher in Greece in comparison with Italy. Significantly higher levels of avoidance-focused coping and lower levels of problem-focused coping were found in the UK sample compared to the other countries. The Italian sample reported higher emotion-focused coping compared to the UK sample.

**Table 7:** Group Means and ANOVA Tests

Means and differences	Total Sample	Greece (1)	Italy (2)	UK (3)	*F* Statistic	Post Hoc
Technostress Factors						
Techno-overload	10.74 (4.56)	10.89 (4.65)	10.29 (4.41)	10.75 (4.53)	0.718	—
Work-home conflict	11.97 (4.94)	11.73 (5.05)	10.92 (4.72)	12.55 (4.85)	5.267*	3 > 2
Techno-ease	16.22 (3.76)	16.30 (3.76)	15.18 (3.67)	16.50 (3.79)	5.383*	1 > 2 3 > 2
Techno-reliability	14.01 (3.70)	13.78 (3.75)	13.52 (3.32)	14.37 (3.76)	3.148*	—
Techno-sociality	12.07 (2.46)	11.82 (2.86)	12.06 (2.10)	12.29 (2.14)	2.909	—
Pace of change	14.14 (4.30)	14.62 (4.77)	13.19 (3.64)	14.04 (3.97)	4.922*	1 > 2
COPE Inventory						
Problem-focused coping	56.61 (18.27)	58.32 (17.27)	59.31 (13.53)	54.41 (20.00)	5.726*	1 > 3 2 > 3
Avoidance-focused coping	35.93 (11.13)	35.97 (9.54)	32.49 (8.37)	37.77 (12.67)	12.727**	3 > 1 3 > 2
Emotion-focused coping	27.64 (10.77)	28.23 (11.49)	29.59 (9.15)	26.57 (10.52)	4.455*	2 > 3
HADS						
Anxious symptoms	8.81 (6.96)	7.61 (4.37)	8.73 (4.76)	9.92 (4.69)	21.429**	3 > 1 3 > 2
Depressive symptoms	6.99 (3.94)	6.31 (3.69)	6.08 (3.56)	7.76 (4.10)	14.555**	3 > 1 3 > 2

*Note*. From left to right, Mean(SD), F statistic and Bonferroni Post Hoc. 1 = Greece, 2 = Italy, 3 = UK.

*p < .05. **p < .001.

### Pearson’s Correlations and Regression Analyses

Table 8 shows the Pearson correlation coefficient matrix for the study variables. In regard to hypotheses 1 and 2, significant associations were found between techno-overload (*r* = .241, *p < .*001), work-home conflict (*r* = .350, *p < .*001), techno-ease (*r* = -.214, *p < .*001), techno-reliability (*r* = -.196, *p < .*001), techno-sociality (*r* = -.123, *p =* .001) and depressive symptoms, but no significant correlation was found between pace of change (*r* = -.010, *p* = .795) and depressive symptoms. Significant correlations were found between techno-overload (*r* = .307, *p < .*001), work-home conflict (*r* = .285, *p < .*001), techno-ease (*r* = -.199, *p < .*001), techno-reliability (*r* = -.160, *p < .*001) and anxiety symptoms, but not between techno-sociality (*r* = -.064, *p* = .087), pace of change (*r* = .057, *p* = .122) and anxiety symptoms.

**Table 8:** Pearson’s Correlations Between Study Variables

Variable	1	2	3	4	5	6	7	8	9	10	11	12	13	14	15	16	17
1. Age	—																
2. Sex (Female)	-.078*	—															
3. International Student (Yes)	.060	.029	—														
4. Level of study (Masters)	.230**	-.090*	-.035	—													
5. Employment status (Employed)	.225**	-.055	-.058	.099*	—												
6. Relationship status (Single)	-.327**	-.028	-.050	-.121**	-.062	—											
7. Techno-overload	.013	.101*	.016	-.062	.047	.048	—										
8. Work home conflict	.004	.078*	.012	.019	.128**	.027	.545**	—									
9. Techno-ease	.037	-.138**	-.008	.106*	.026	.001	-.278**	-.189**	—								
10. Techno-reliability	.085*	-.133**	-.040	.078*	-.004	-.071	-.340**	-.273**	.554**	—							
11. Techno-sociality	.021	.011	-.040	.092*	.156**	-.026	-.114*	-.014	.238**	.237**	—						
12. Pace of change	-.042	.043	.014	-.029	.155**	.038	.138**	.092*	-.032	-.054	.235**	—					
13. Problem-focused coping	.064	.106**	.020	.037	-.118**	-.036	.014	-.090*	.100*	.201**	-.080*	-.005	—				
14. Avoidance-focused coping	-.093*	.054	.025	-.009	-.044	.120**	.095*	.095*	-.001	.030	-.070	.027	.494**	—			
15. Emotion-focused coping	.011	.247**	.026	.013	-.180**	-.072*	.089*	-.086*	.023	.121*	-.101*	-.044	.656**	.373**	—		
16. Anxiety symptoms	-.132**	.146**	-.005	-.068	-.019	.037	.307**	.285**	-.199**	-.160**	-.064	.057	-.014	.280**	.189**	—	
17. Depressive symptoms	-.128**	.058	-.036	-.060	.022	.080*	.241**	.350**	-.214**	-.196**	-.123*	-.010	-.179**	.231**	-.045	.620**	—

*p < .05. **p < .001.

Four multiple regression analyses (enter method) were then performed. Two of these included the six technostress factors and demographic variables as predictors and the HADS anxiety and depression subscale as outcomes. The other two included coping factors and demographics as predictors and the HADS subscales as outcomes (Table 9). Model 1 explained 16.9% of the total variance (*p <* .001) in anxiety symptoms. Techno-overload (β = .187, *p <* .001), work-home conflict (β = .201, *p <* .001), techno-ease (β = -.116, *p* = .011) and age (β = -.100, *p <* .001) were significant predictors of anxiety symptoms. Model 2 explained 16.6% of the total variance (*p* < .001) in depressive symptoms. Work-home conflict (β = .290, *p <* .001), techno-ease (β = -.122, *p =* .008) and age (β = -.118, *p =* .004) were significant predictors of depressive symptoms. Model 3 explained 17.1% of the total variance in anxiety symptoms (*p* < .001), and problem-focused coping (β = -.290, *p <* .001), emotion-focused coping (β = .221, *p <* .001), avoidance-focused coping (β = .327, *p <* .001) and sex (female) (β = .089, *p =* .017) were significant predictors of anxiety symptoms. Model 4 explained 14.5% of the variance (*p <* .001) in depressive symptoms. Problem-focused coping (β = -.312, *p <* .001) and avoidance-focused coping (β = .317, *p <* .001) were significant predictors of depressive symptoms. All regression models met multicollinearity and error independence assumptions (Table 9).

**Table 9:** Regression Analyses For the HADS Anxiety and Depression Subscales

	Anxiety Symptoms		Depressive Symptoms	
Predictor	Statistic	*p* value	Statistic	*p* value
Regression model	Model 1		Model 2	
	*R*^2^ = .169	< .001	*R*^2^ = .166	< .001
	*D-W =* 1.809		*D-W =* 1.987
	*VIF =* 1.1		*VIF =* 1.1
Age	β = -.100	< .001	β = -.118	.004
Sex (Female)	β = .058	.124	β = -.019	.621
International student (Yes)	β = .012	.726	β = .000	.001
Level of study (Masters)	β = -.028	.464	β = -.034	.381
Employed (Yes)	β = .030	.427	β =.049	.196
Relationship status (Single)	β = -.028	.460	β =.008	.840
Techno-overload	β = .187	< .001	β = .030	.517
Work-home conflict	β = .201	< .001	β = .290	< .001
Techno-ease	β = -.116	.011	β = -.122	.008
Techno-reliability	β = .017	.714	β = -.025	.604
Techno-sociality	β = .038	.346	β = -.043	.287
Pace of change	β = -.006	.873	β = -.056	.143
Regression model	Model 3		Model 4	
	*R*^2^ = .171	< .001	*R*^2^ = .145	< .001
	*D-W =* 1.829		*D-W =* 1.956	
	*VIF =* 1.2		*VIF =* 1.3	
Age	β = -.047	.227	β = -.045	.265
Sex (Female)	β = .089	.017	β = .059	.118
International student (Yes)	β = .017	.640	β = -.007	.856
Level of study (Masters)	β = -.052	.161	β = -.043	.250
Employed (Yes)	β = .051	.160	β = .037	.324
Relationship status (Single)	β = -.047	.211	β = -.015	.687
Problem focused coping	β = -.290	< .001	β = -.312	< .001
Emotion-focused coping	β = .221	< .001	β = .049	.279
Avoidance coping	β = .327	< .001	β = .317	< .001

*Note.* β: standardised beta. D-W: Durbin-Watson value. VIF: Variance Inflation Factor value.

### Coping as a Mediator Between Technostress Factors and Anxiety Symptomatology

Multiple mediation analysis was used to test hypothesis 3. The first mediation analysis investigated coping as a mediator between techno-stress factors and anxiety symptoms (Table 10). The total effect of techno-overload on anxiety symptoms was significant, β = .179, 95% CI [.080, .272]. Techno-overload had a significant indirect effect through problem-focused coping, β = -.034, 95% CI [-.077, -.002], which accounted for 18.99% of the total effect of techno-overload on anxiety symptoms. In addition, techno-overload had a significant indirect effect through emotion-focused coping, β = .031, 95% CI [.011, .060], which accounted for 17.32% of the total effect of techno-overload on anxiety symptoms. No evidence for an indirect effect was found between techno-overload and anxiety symptoms through avoidance coping.

**Table 10:** Outcomes of Multiple Mediation Analyses (Bootstrapped 5000 Samples)

			Total Effect			Direct Effect			Effect of IV on M	Effect of M on DV	Indirect Effect		
Outcome	Predictor	Mediator	*β*	*SE*	CI	*β *	*SE*	CI			*β *	*SE*	CI
Anxiety	Techno-overload		.179**	.044	.080, .272	.163**	.041	.069, .254					
		Problem-focused							.111*	-.252**	-.034*	.018	-.077, -.002
		Avoidance coping							.066	.264**	.018	.017	-.015, .058
		Emotion-focused							.202**	.232**	.031*	.012	.011, .060
	Work-home conflict		.207**	.043	.116, .307	.179**	.041	.089, .268					
		Problem-focused							-.131*	-.244**	.015	.017	-.024, .055
		Avoidance coping							.064	.251**	.027*	.017	.001, .066
		Emotion-focused							-.188**	.277**	-.014	.001	-.041, .007
	Techno-ease		-.087*	.037	-.159, -.010	-.078*	.035	-.150, -.007					
		Problem-focused							.103*	-.246**	-.043*	.016	-.079, -.016
		Avoidance coping							.026	.280**	.016	.014	-.011, .049
		Emotion-focused							.041	.252**	.018*	.009	.003, .040
Depression	Work-home conflict		.317**	.036	.243, .390	.284**	.035	.211, .354					
		Problem-focused							-.074*	-.244**	-.001	.014	-.032, .028
		Avoidance coping							.097*	.266**	.034*	.014	.007, .066
	Techno-ease		-.156**	.037	-.233, -.081	-.131**	.035	-.204, -.055					
		Problem-focused							.084*	-.244**	-.038*	.015	-.073, -.011
		Avoidance coping							.015	.298**	.013	.014	-.013, .043

*Note*. β: standardised beta. *SE*: standard error. CI: bias corrected accelerated 95% confidence intervals.

The total effect of work-home conflict on anxiety symptoms was significant, β = .207, 95% CI [.116, .307]. Work-home conflict had a significant indirect effect through avoidance coping, β = .027, 95% CI [.001, .066] that accounted for 13.04% of the total effect of work-home conflict on anxiety symptoms. No evidence for an indirect effect was found between work-home conflict and anxiety symptoms through problem- or emotion-focused coping.

The total effect of techno-ease on anxiety symptoms was also significant, β = -.087, 95% CI [-.159, -.010], with indirect effects through problem-, β = -.043, 95% CI [-.079, -.016] and emotion-focused coping, β = .018, 95% CI [.003, .040] accounting for 49.43% and 20.69% of the total effect, respectively. No evidence for an indirect effect was found between techno-ease and anxiety symptoms through avoidance coping. The residual direct effects for techno-overload, β = .163, 95% CI [.069, .254], work-home conflict, β = .179, 95% CI [.089, .268] and techno-ease, β = -.078, 95% CI [-.150, -.007] on anxiety symptoms indicated partial mediation (Table 10).

### Coping as a Mediator Between Technostress Factors and Depressive Symptomatology

Multiple mediation analysis was performed to investigate coping style as a mediator between technostress factors and depressive symptoms (Table 10). The total effect of work-home conflict on depressive symptoms was significant, β = .317, 95% CI [.243, .390]. Work-home conflict had a significant indirect effect through avoidance coping, β = .034, 95% CI [.007, .066], which accounted for 10.73% of the total effect of work-home conflict on depressive symptoms. No evidence for an indirect effect was found between work-home conflict and depressive symptoms through problem-focused coping.

The total effect of techno-ease on depressive symptoms was significant, β = -.156, 95% CI [-.233, -.081], with an indirect effect through problem-focused coping, β = -.038, 95% CI [-.073, -.011] accounting for 24.36% of the total effect. No evidence for an indirect effect was found between techno-ease and depressive symptoms through avoidance coping. The residual direct effects for work-home conflict, β = .284, 95% CI [.211, .354] and techno-ease, β = -.131, 95% CI [-.204, -.055] on depressive symptoms indicated partial mediation (Table 10).

## Discussion

This study investigated the associations between techno-stressors, coping, and anxious and depressive symptoms in university students during an intensive period of technology usage. Universities across the globe had to adapt quickly to deliver their courses during the COVID-19 pandemic and it is anticipated that reliance on technology in HE will last for the foreseeable future (Bloomfield, 2020). Understanding how technostress translates into psychopathological outcomes in the student population is therefore important to support students in facing the heightened ICT challenges introduced by the pandemic.

The study found that work-home conflict was associated with greater anxiety and depressive symptoms. This has been found in previous research, which showed that greater work-home conflict exists when university work and personal life are integrated rather than separated (Adebayo, 2006; McCutcheon & Morrison, 2018). Stricter boundaries between technology, work, and personal life may allow students to mentally detach from their work and protect them against anxiety and depression.

A substantial body of research has investigated how workers cope with managing the boundaries between their work and home life, and how this relates to psychopathology (e.g., Bergs et al., 2018; McTernan et al., 2016). The results of the current study show a direct effect of work-home conflict on anxiety and depressive symptoms as well as an indirect effect through avoidance coping. Considering the specific context of the pandemic and lockdown, the use of avoidance coping to manage conflict between work/home life may have resulted in students closing themselves off and/or hiding into their ICT activities, which, in turn, increased their anxiety and depressive symptoms.

Previous research shows that dealing with the complexity of technology and/or the uncertainty that comes with constant changes, developments, and upgrades in ICT can lead to stress, anxiety, and depression (Dragano & Lunau, 2020; Thomée, 2012). It is now more essential than ever that students renew their technical skills while dealing with the pressure of more complex systems and virtual learning environments. The findings of the present study reveal a negative association between techno-ease and anxiety and depressive symptoms. Techno-ease had a protective direct effect on anxiety and depressive symptoms in addition to an indirect effect through problem-focused coping. Techno-ease and problem-focused resolution can be supported by institutions providing their students with accessible ICT services, training, and workshops, as well as clear online ICT instructions and resources.

Techno-overload was positively associated with anxiety and depressive symptoms, which is in line with previous research on general population samples (Gaudioso et al., 2017). The mediation analysis suggested that when techno-overload is high, the indirect effect of problem-focused coping protected against anxiety, whereas the indirect effect of emotion-focused coping increased anxiety symptoms. This latter finding contradicts previous research, which suggests that emotion-focused resolution through social support, including chatting with friends/family online, translates into positive outcomes for wellbeing (Liu et al., 2018; Zhu et al., 2013). One explanation for our finding could be situational factors since access to support networks during the data collection period would likely have been through ICTs due to social restriction measures. Engaging in emotion-focused coping during this period could therefore have contributed to increased techno-overload, necessitated intensive screen time, and resulted in a bi-directional relationship between these variables that resulted in heightened student anxiety. This is supported by research on Facebook Addiction Disorder (FAD), which showed that individuals who received high levels of social support online were at risk for tendencies toward FAD and that this negatively influenced mental health (Brailovskaia et al., 2019). Furthermore, another aspect of ICT is that communication can occur via several channels simultaneously (e.g., webchats, mobiles, video calls, etc.), which can be mentally exhausting and potentially stressful since distractions and dual tasking are demanding on working memory (Nijboer et al., 2016). With this in mind, access to social support through ICT during a period in which reliance on ICT was already high may have contributed toward heightened anxiety symptoms in this sample. However, this is somewhat speculative given the cross-sectional nature of the current research, and longitudinal studies will be needed to confirm this hypothesis.

An interesting finding in the present study was that significantly higher levels of anxiety and depression were found in the UK sample compared to Italy and Greece. Higher levels of avoidance coping and lower levels of problem-focused coping were also found in the UK sample compared to the other countries, and work-home conflict was significantly higher in the UK compared to Italy. Techno-ease was significantly lower in the Italian sample compared to the other countries, and pace of change was significantly higher in Greece in comparison to Italy. Students in Italy reported significantly higher emotion-focused coping compared to the UK. These observed differences could be due to a wide variety of factors, including individual differences in socio-cultural factors, pandemic specific responses within countries, or differences in the academic environment/demands among the participating countries. Although these differences between the countries are interesting, they should be interpreted with caution. We did not confirm measurement invariance on the COPE and HADS, limiting the conclusions that can be made regarding statistical differences on these variables. However, the instruments have previously been validated in the respective countries, which supports their use in a range of populations (Anastasiou et al., 2017; Coriale et al., 2012; Ferrandina et al., 2012) including students (Fradelos et al., 2019; Sagone & De Caroli, 2014). Further, more research is needed in order to specify the exact factors and underlying mechanisms that may account for these differences at a country-level.

The overall sample for the current study was relatively young (*M =* 21.58, *SD* = 4.29). Although this is reflective of the broader student population, it is difficult to generalise our findings to mature learners. Hauk et al. (2019) found that even though older people are more prone to techno-stressors, ageing is connected to development of coping skills that in turn help reduce negative outcomes of technostress. However, increased home/work conflict is more common for mature learners (Markle, 2015; van Rhijn et al., 2016), as these students often experience greater social and family responsibilities. Future research could therefore extend our paradigm to establish whether these relationships are also present in mature student samples. Another limitation is that the primary language of the study participants was not assessed. We worked on the assumption that students had sufficient proficiency in the language of the country in which they were studying. Although we did measure the status of international students in our design, which may have accounted for non-native speakers to some extent, this working assumption could have affected the results.

Finally, it should also be noted that technostress can act as an “enhancer” to one’s productivity (Hung et al., 2015), therefore possibly giving some users the perception that while they are working faster and longer with their ICTs, they are also working more efficiently. It is possible that while technostress may have increased anxiety and depressive symptoms in the students, perceived productivity could also have resulted in the experience of positive feelings, such as accomplishment, which may serve as a protective factor. Although we did include some positive effects of technology in our design (techno-sociality, techno-ease, techno-reliability), we did not account for other possible benefits of technology and acknowledge this as a further limitation of the study.

## Conclusions

The current study investigated associations between technostress, coping, and anxiety/depressive symptoms in European university students during disruption to the higher education sector caused by the COVID-19 pandemic. Further data and psychological interventions are needed to promote psychological health among students in the immediate future and also after the pandemic. The psychological consequences of the COVID-19 outbreak will unfortunately last. An understanding of how technostress translates through coping strategies into mental health outcomes can help student counselling centres target maladaptive coping strategies, thus providing appropriate support to students.
